# Possible Approach to Esophageal Lung with Long Tracheobronchial Gap

**DOI:** 10.1055/s-0039-1692407

**Published:** 2019-06-17

**Authors:** Martina Ichino, Lorenza Pugni, Andrea Zanini, Anna Morandi, Fabio Mosca, Francesco Macchini

**Affiliations:** 1 Department of Pediatric Surgery, Fondazione IRCCS Ca' Granda, Ospedale Maggiore Policlinico, Milano, Italy; 2 Department of Neonatal Intensive Care Unit, Fondazione IRCCS “Ca' Granda” Ospedale Maggiore Policlinico, Milano, Italy

**Keywords:** esophageal lung, pneumonectomy, thoracoscopy

## Abstract

Esophageal lung is a rare bronchopulmonary foregut malformation characterized by an anomalous origin of one of the main bronchi which arises from the esophagus. Less than 30 cases are reported in the literature. Therefore, there are no standardized guidelines for the treatment of this condition. We report a case of right esophageal lung diagnosed in a neonate. The patient was treated with thoracoscopic closure of the ectopic main bronchus in the neonatal period, followed by delayed pneumonectomy at 5 months of age. No prosthetic substitute was implanted in the ipsilateral hemithorax after pneumonectomy. The patient is now 4 years old and doing well, postpneumonectomy syndrome was never observed. Our strategy and the possible alternatives are discussed here.

## Introduction

Esophageal lung is an extremely rare malformation characterized by an anomalous origin of one of the main bronchi, which arises from the esophagus. Only few cases have been reported in the literature and no standardized guidelines exist for its treatment. 1

We report a case of right esophageal lung diagnosed in a neonate and discuss our management and possible alternative treatment strategies.

## Case Report

A female newborn was referred to our center from a peripheral hospital on day 2 of her life after onset of respiratory distress, right atelectasis at the chest X-ray, and elevation of inflammatory indexes. She was delivered at 37 ^5/7^ weeks of gestation by an elective cesarean section for maternal indications. The pregnancy was uneventful apart from the suspect of fetal dextrocardia.

At admission she presented mild dyspnea and breath sounds were present only in the left hemithorax, while heart sounds could be heard on the right hemithorax. A chest X-ray confirmed the right atelectasis and no cardiac shadow was visible in the left hemithorax. Echocardiography revealed a right cardiac shift rather than dextrocardia and a severely hypoplastic right pulmonary artery.

The newborn required noninvasive ventilation with nasal continuous positive airway pressure (nCPAP) with FiO _2_ 0.21 to maintain an arterial oxygen saturation of 98% with a good respiratory dynamic.

**Fig. 1:**
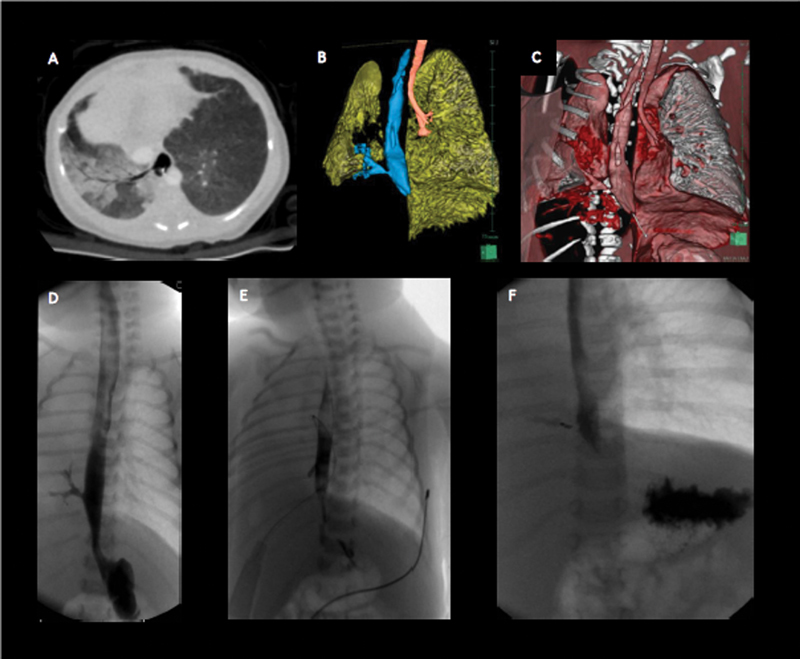
Upper row: CT scan on day 3 of life. ( **A** ) Transverse section showing the anomalous origin of the right bronchus; ( **B** , **C** ) three-dimensional anatomy reconstruction showing the trachea ending with the left bronchus in the left lung and the right bronchus originating from the esophagus. Lower row: esophageal contrast studies ( **D** ) Esophageal contrast study on day 3 of life. The oral contrast evidences the anomalous origin of the right bronchus from the esophagus. ( **E** ) Contrast study on the 3 ^rd^ post-operative day after closure of the anomalous bronchus with clips. ( **F** ) Evidence of partial re-canalization of the anomalous bronchus at 5 months of age. CT, computed tomography.

On day 3 of her life, a chest computed tomography (CT) scan ( Fig. 1A–C ) was performed and revealed an anomalous origin of the right main bronchus from the distal esophagus at the level of T9, while the trachea ended directly into the left main bronchus at the level of T4. The CT scan also confirmed the presence of severe right pulmonary artery hypoplasia. The right lung was partially air filled possibly after retrograde air inflation from the digestive tract, mainly due to the nCPAP. An associated pulmonary airway malformation could not be excluded by the CT scan. The diagnosis of right esophageal lung was made and confirmed by an esophageal contrast study performed on the same day ( Fig. 1D ).

On day 4 of her life, the patient was taken to the operating theater for endoscopic examination of airways and esophagus to better characterize the anatomy and make a treatment plan. The tracheobronchoscopy showed no signs of tracheomalacia, the carina was absent, and the trachea ended directly into the left main bronchus. Esophagoscopy showed a tubular cartilaginous structure arising from the right wall of the distal third of the intrathoracic esophagus.

We decided to proceed with a thoracoscopic approach straight after the endoscopic examination. The aim was closing the anomalous bronchus to avoid pneumonia, while leaving the lung in place and delaying the pneumonectomy later in time to reduce the risk of acute mediastinal shift and consequent postpneumonectomy syndrome. 2

**Fig. 2:**
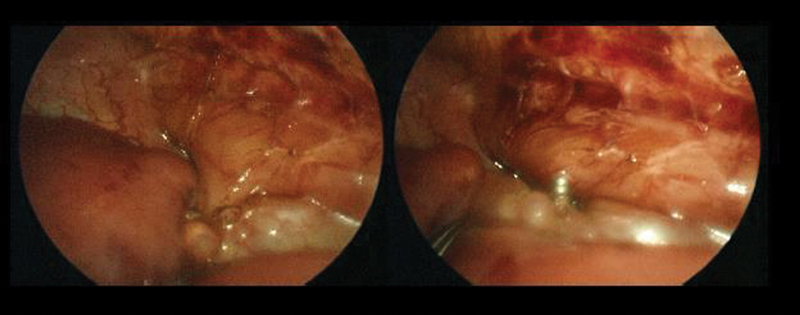
Intraoperative images of the anomalous bronchus arising from the esophagus before and after ligation with titanium endo-clips.

The neonate was placed on her left decubitus and three trocars were placed: a 3 mm one for the camera was placed in the 5th intercostal space below the tip of the scapula, two operative ports were placed—a 5 mm one on the 6th intercostal space on the posterior axillary line, and a 3 mm one on the 4th intercostal space on the anterior axillary line. Almost complete pulmonary atelectasis and right lung infarction was evident. A window in the mediastinal pleura was created and the esophageal bronchus arising from the right wall of the distal esophagus was detected. The anomalous bronchus was isolated and closed with two 5 mm titanium endoclips ( Fig. 2 ).

The immediate postoperative course was uneventful. On the 3rd postoperative day, after a negative esophageal contrast study ( Fig. 1E ), feeding was started and well tolerated. We decided to perform an early contrast study to confirm the complete interruption of the aerodigestive communication to start an early oral feeding. The patient was discharged on the 15th postoperative day with an antibiotic prophylaxis and a regular follow-up program. We scheduled the pneumonectomy after 6 months of life.

In the following months she presented twice with fever that was successfully treated with antibiotics. At the third episode, at 5 months of age, she was admitted to our neonatal intensive care unit (ICU). An esophageal contrast study was performed which revealed a partial recanalization of the right main bronchus with a small leakage toward the right lung past the endoclips ( Fig. 1E ). Chest X-ray showed a relevant cardiac shift to the right side despite the native lung was still in the thoracic cavity; however, the patient didn't present symptoms related to mediastinal shift. Therefore, we decided to proceed with the pneumonectomy.

A right posterolateral thoracotomy was made. The pulmonary hilum was isolated, and the pulmonary artery and veins were ligated and divided. The ectopic right bronchus was then identified and divided at its origin. The esophageal wall was repaired with an interrupted single–layer, long-term absorbable suture. Considering the absence of symptoms, despite the presence of mediastinal shift, no thoracic expander was placed into the chest. A chest drain was left in situ.

The postoperative course was characterized by a minor esophageal leakage diagnosed by a contrast study performed on the 6th post-operative day due to salivary leak from the chest drain. It was successfully treated conservatively with fasting and antibiotics. Histological examination of the right lung revealed complex pulmonary airways malformation with squamous metaplasia of segmental bronchial epithelium and histiocytosis of locoregional lymphnodes.

A multidisciplinary follow-up was organized comprehensive of regular visits in our thoracic surgery outdoor clinic and in the follow-up service of the neonatal unit. The patient is now 5 years old and has a normal life, needing respiratory physiotherapy cycles for high airways resistance.

## Discussion

In case of esophageal lung, interrupting the communication between the esophagus and the bronchus is essential to allow oral intake without developing airway infections. The subsequent surgical options include either the connection of the unventilated lung to the respiratory tree through a bronchotracheal anastomosis, or a pneumonectomy. In most case report, the treatment of choice is pneumonectomy, 1 2 3 4 5 6 7 8 but there are reports of successful tracheobronchial reconstructions. 9 10 11 In our case, we judged that a tracheobronchial anastomosis was unfeasible due to the long tracheobronchial gap, in fact the position of the right main bronchus was very low in the thorax with a gap of four vertebral bodies between its origin and the end of the trachea, as it is well evidenced by the three-dimensional CT reconstruction of the anatomy of our patient ( Fig. 1B–C ). Furthermore, the patient presented severe right pulmonary artery hypoplasia that represented a contraindication to the preservation of the lung. For these reasons we decided to perform the pneumonectomy.

Concerning the timing, we were concerned of a possible postpneumonectomy syndrome which, although rare, is caused by mediastinal shift leading to kinking of the trachea and of the main heart vessels with respiratory and circulatory consequences. The literature concerning this dangerous complication is scant, consisting mainly of case reports. Fatal cases are reported after pneumonectomy in infants 12 13 and severe mediastinal shift is especially related to right pneumonectomy due to the normal mediastinal anatomy with a left-sided aortic arch. 14 15 Even if not definitively demonstrated, the risk of postpneumonectomy syndrome is considered to be higher in the pediatric population 15 due to the softer mediastinal connective tissue and consequently even higher in the neonates. It is on this basis that we decided to postpone the pneumonectomy and preserved the native lung as a natural expander to eliminate the need of prosthetic material. Initial closure of the bronchus, delayed pneumonectomy, and administration of antibiotic prophylaxis allowed us to reach our therapeutic goals: feeding, prevention of pneumonias, and prevention of the complication, following pneumonectomy. Nonetheless, the patient presented with pneumonia after the first operation. The right main bronchus was clipped but not divided. The infective episodes were a consequence of a reestablished communication between the esophagus and the right lung. There are no reports about partial recanalization in esophageal lung, since there are no case reports of a “two-stage” approach to this malformation. However, dislocation of metal clips have been reported in tracheoesophageal fistula (TEF) repair. 16 Shortly after the presented case, we had a case of isolated TEF that we closed with metal clip and that developed a recurrence of the fistula, following clip dislocation. For these reasons, from that moment on we started using self-locking clips such as “Hem-o-lok ^®^ ” or transfixed suture and division of the fistula with scissor and up to now we haven't had other similar complications. However, in the presented case, the recanalization occurred without any clip dislocation, as assessed during the second operation. Therefore, retrospectively, we think that closing the bronchus with a self-locking clip, dividing it at its origin from the esophagus, and suturing the esophageal wall might have prevented the recurrent infections. Once we evidenced the partial recanalization of the anomalous bronchus, and as the chest X-ray showed an important mediastinal shift despite the presence of the native lung, the decision to proceed with the pneumonectomy without placing a thoracic expander seemed reasonable, and, at present, with a 4-year follow-up, the patient is doing well.

One can claim that an early pneumonectomy could have prevented the recurrent infections determining a definitive treatment in only one step, but we cannot state that the native lung failed in preventing early postpneumonectomy syndrome; it may have been effective by avoiding the immediate shift of the mediastinum and allowing a slow adaptation to the new anatomy.

## Conclusion

The management of esophageal lung is challenging. We believe that, if feasible, the treatment of choice should be the tracheobronchial anastomosis with the preservation of the lung to allow the best quality of life to the affected child. However, if this approach is not possible for anatomical reasons, such as a long trachealbronchial gap, vascular anomalies, or pulmonary malformation, in those cases, we believe that a reasonable treatment plan is an early mini-invasive closure and division of the esophageal bronchus, followed by delayed pneumonectomy of the esophageal lung. This allows a slow adaptation of thoracic anatomy to reduce the risk of postpneumonectomy syndrome.

## Notes

New Insights and the Importance for the Pediatric Surgeon The management of esophageal lung is a challenging subject. The proposed minimally-invasive, two-step approach may be of some usefulness in cases of esophageal lung with long tracheobronchial gap and associated pulmonary and vascular anomalies.
